# Development and Validation of the Postpartum Quality of Sexual Life Questionnaire: A Prospective Observational Cohort Study

**DOI:** 10.7759/cureus.83466

**Published:** 2025-05-04

**Authors:** Dragos Brezeanu, Ana-Maria Brezeanu, Traian Surdu, Vlad I Tica

**Affiliations:** 1 6th Department, Ovidius University, Constanta, ROU; 2 2nd Department, Ovidius University, Constanta, ROU

**Keywords:** pregnancy, puerperium, quality of sexual life, questionnaire, sexual function

## Abstract

Background: The postpartum period, which is defined as the six to eight weeks following childbirth, is characterized by significant physical, emotional, and hormonal changes, potentially impacting the quality of sexual life in women. Although postpartum sexual health is critical for overall quality of life and relationship stability, influencing factors remain insufficiently explored. This study aims to identify and analyze the influence of delivery mode, maternal age, educational level, and parity on postpartum sexual function and quality of life.

Methods: A prospective observational cohort study was conducted at Saint Andrew Hospital, Constanta, Romania, between February 1, 2023, and December 31, 2023. A total of 100 women, 40-60 days postpartum, completed the newly developed Postpartum Quality of Sexual Life Questionnaire (PPQSL-Q), adapted from the validated Pelvic Organ Prolapse Urinary Incontinence Sexual Function Questionnaire-12. Participants were categorized by birth modality, age group, educational level, and parity. Statistical analysis included descriptive statistics (frequencies, percentages, means, and standard deviations), independent samples t-tests, ANOVA, and Pearson correlation coefficients to examine relationships between questionnaire scores and participant variables. Statistical significance was set at a p-value of <0.05.

Results: Cesarean births were significantly associated with higher postpartum sexual quality-of-life scores compared to spontaneous births (mean score 42.22 vs. 40.74, p < 0.01). Educational level demonstrated a moderate positive correlation (*r* = 0.211), with participants having middle-level education showing the highest mean scores (42.94). Age and parity showed weak but positive correlations (*r* = 0.056 and* r* = 0.157, respectively), with older women (33-39 years) and multiparous women reporting slightly better sexual function scores.

Conclusion: Mode of delivery significantly influences postpartum sexual quality of life, while educational level, age, and parity present moderate to mild correlations. These findings underline the need for tailored postpartum counseling and care strategies to support women's sexual health recovery.

## Introduction

The World Health Organization (WHO) has defined sexual health as a state of overall well-being, encompassing physical, emotional, mental, and social aspects of sexuality [1]. It is a fundamental element of human life, contributing significantly to one's quality of life and well-being. The period surrounding childbirth and the transition into parenthood can be a stressful time for many couples, affecting both their health and quality of life. It's important to note that while giving birth, particularly vaginally, can have a lasting impact on postpartum quality of life, maintaining a healthy sexuality remains an essential aspect of life [2].

The postpartum period, called puerperium, is a pivotal and transformative phase that spans six to eight weeks after childbirth [3]. Significant physical, emotional, and hormonal changes occur as the body recovers and adjusts to no longer being pregnant. This period is crucial for both mother and newborn, as it marks a time of intense physical and emotional changes, recovery, and adjustment to parenthood [4].

New parents, caregivers, and healthcare providers must comprehensively understand the postpartum period to ensure the optimal health and happiness of both the mother and the child [5].

During the postpartum period, particularly within the first three months following delivery, it is common for women to experience some form of sexual dysfunction [6].

Studies have shown that 35% to 83% of women are affected. While many women may resume sexual activity within the first three months after giving birth, 83% of them report experiencing sexual problems. While the percentage drops to 64% in the first six months, a significant number of women (38%) are unable to enjoy the same quality of sexual life as before pregnancy [7]. Researchers have identified several factors that can contribute to sexual dysfunction, including primiparity, age, mode of delivery, education, family income, partnership issues, and exclusive breastfeeding [8].

After giving birth, a woman's body goes through a series of significant changes as it begins to heal and return to its pre-pregnancy state. One of the most immediate changes is the reduction of the uterus size, a process known as involution. Initially weighing about 1000 grams, the uterus quickly shrinks to 50-100 grams within six weeks, often accompanied by "afterpains" that help minimize bleeding [9].

Breastfeeding presents its own set of changes and challenges. It stimulates the production of prolactin, which aids in milk production, and oxytocin, which helps the uterus contract [10]. However, breastfeeding can also cause pain and discomfort, leading to issues such as engorgement, blocked milk ducts, and mastitis [11]. Proper latching techniques and consulting lactation experts can help alleviate many of these concerns [10].

Physical recovery also involves dealing with lochia, the vaginal discharge composed of blood, mucus, and uterine tissue that can last for up to six weeks [3]. During this time, patients must monitor their health for signs of complications, such as infections or excessive bleeding. Moreover, many women experience significant fatigue due to the demands of newborn care, hormonal adjustments, and the physical recovery process [12]. Postpartum women should prioritize rest, nutrition, and support to aid in their physical recovery.

During the postpartum period, the body experiences several hormonal changes that can affect sexual desire and physical readiness for sexual activity [13]. The decrease in estrogen and progesterone levels, coupled with elevated prolactin levels for breastfeeding, can lead to vaginal dryness and decreased libido [14]. Additionally, the physical healing process, especially if there was perineal tearing or a cesarean section, requires time, which can affect when sexual activity can be comfortably resumed [15].

New mothers may experience various emotional changes and obstacles during the postpartum period. While they may feel joy and bonding with their babies, they may also experience anxiety and fear about the changes in their lives and bodies [16]. These emotional fluctuations, as well as concerns about body image after childbirth, can complicate the resumption of a fulfilling sexual life [17].

It is important to note that the timing for resuming sexual activity varies among individuals, and medical professionals generally advise waiting until postpartum bleeding has stopped and any tears or incisions have healed. Couples should communicate openly about their readiness, desires, and any concerns they may have. Both partners should practice understanding and patience to navigate this transition successfully [18].

To alleviate physical discomfort during sex, using lubricants to address vaginal dryness and experimenting with pain-free positions can be beneficial. In cases of persistent sexual or emotional issues, seeking guidance from a healthcare provider or therapist can provide effective coping strategies and improve the situation [19]. In many countries, mothers often provide valuable guidance during such conditions, offering advice on what actions should be taken and how to manage the situation scientifically, instead of relying solely on a healthcare provider [16].

We were exploring the various factors that can impact sexual life after childbirth, a crucial topic for new parents as it can significantly affect their well-being and relationships [20].

This study aimed to investigate the different factors that influence the sexual lives of young parents and can have significant effects on their well-being and relationships.

## Materials and methods

We conducted a prospective cohort observational study at the Saint Andrew Hospital, Constanta, Romania, between February 1, 2023, and December 31, 2023. Ethical approval was obtained from the Ethics Commission of Saint Andrew Hospital, Constanta (approval 01/20.01.2023).

The questionnaire was developed after thorough consultation with experts, a comprehensive literature review, and adaptation of previously validated instruments evaluating sexual function. Specifically, we adapted the Pelvic Organ Prolapse/Urinary Incontinence Sexual Function Questionnaire-12 (PISQ-12) [21-23]. The adaptation involved translating the original English-language PISQ-12 into Romanian and modifying it for relevance in postpartum women. The resulting questionnaire, titled "Postpartum Quality of Sexual Life Questionnaire" (PPQSL-Q), consisted of 12 behavioral, emotional, physical, and partner-related items assessed on a 5-point Likert scale from ‘never’ (1) to ‘always’ (5). Items 1-4 were reverse-scored, yielding a maximum possible score of 48. The Romanian version was subsequently translated into English (Table 1).

**Table 1:** English-translated Postpartum Quality of Sexual Life Questionnaire (PPQSL-Q)

Postpartum Quality of Sexual Life Questionnaire (PPQSL-Q)						
1. How often do you feel the urge to have sex? (May include desire for intercourse, mental planning for intercourse, frustration at not having intercourse):						
Always	Usually	Sometimes		Rarely		Never
2. How often you reach orgasm after sexual contact with your partner?						
Always	Usually	Sometimes		Rarely		Never
3. Do you feel aroused during sexual intercourse with your partner?						
Always	Usually	Sometimes		Rarely		Never
4. How satisfied are you with the sexual acts performed?						
Always	Usually		Sometimes	Rarely	Never	
5. Do you experience pain during sexual intercourse with your partner?						
Always	Usually		Sometimes	Rarely	Never	
6. Do you feel dryness during sexual intercourse with your partner?						
Always	Usually		Sometimes	Rarely	Never	
7. Does the feeling of dryness during sexual intercourse with your partner cause you to have intercourse less frequently compared to intercourse before birth?						
Always	Usually		Sometimes	Rarely	Never	
8. Do you try to avoid sexual intercourse compared to the sexual contacts you had before the birth?						
Always	Usually		Sometimes	Rarely	Never	
9. During intercourse, do you experience negative emotions, such as disgust, fear, guilt, or shame, more than before you gave birth?						
Always	Usually		Sometimes	Rarely	Never	
10. During intercourse, does the partner have problems maintaining an erection, compared to intercourse before birth?						
Always	Usually		Sometimes	Rarely	Never	
11. During intercourse, does the partner have problems with premature ejaculation, compared to intercourse before birth?						
Always	Usually		Sometimes	Rarely	Never	
12. Are the orgasms obtained more intense, compared to sexual contacts before birth?						
Always	Usually		Sometimes	Rarely	Never	

Eligible participants were recruited from women attending postpartum visits at Saint Andrew Hospital. Inclusion criteria were women aged between 18 and 39 years, in heterosexual relationships, capable of reading Romanian, who gave birth 40 to 60 days before study enrollment. Exclusion criteria included preterm or post-term births (<37 or >42 weeks of gestation), high-risk pregnancies requiring specialized care, known congenital anomalies in the newborn, and maternal medical conditions necessitating elective cesarean section (e.g., placenta previa, significant cardiac conditions).

Participants provided informed consent as required by the Saint Andrew Hospital Ethics Commission, Constanta. Participants (n=100) were randomly selected from hospital birth records using a random number generator available at randomisation.org. The sample included an equal distribution of women who had spontaneous vaginal births (n=50) and cesarean sections (n=50) (Table 2).

**Table 2:** Distribution of the studied group by way of birth. Patients were divided into two categories: spontaneous and cesarean section

Way of Birth	Number (%)
Spontaneous	50 (50)
Cesarean Section	50 (50)

We categorized participants into three age groups (18-25, 26-32, 33-39 years) (Table 3), by educational level into Lower (primary classes education), Middle (high school education), and higher (college or university) (Table 4), and by parity (primipara, secundipara, and multipara) (Table 5).

**Table 3:** Distribution of the studied group by age. The patients were divided in three groups: one group between 18 and 25 years old, one group between 26 and 32 years old and one group between 33 and 39 years old.

Way of Birth	Age		
	18-25 (%)	26-32 (%)	33-39 (%)
Spontaneous	18	15	17
Caesarean	11	17	22
Total	29 (29)	32 (32)	39 (39)

**Table 4:** Distribution of the studied group by educational level. The patients were divided in three groups: a lower level of education, a middle level of education and a higher level of education.

Educational level	Lower (%)	Middle (%)	Higher (%)
Spontaneous	15	18	17
Caesarean	10	17	23
Total	25 (25)	35 (35)	40 (40)

**Table 5:** Distribution of the studied group by number of births. The patients were divided into three groups: primipara (first pregnancy), secundipara (second pregnancy) and multipara (three or more pregnancies)

Number of Births	Primipara (%)	Secundipara (%)	Multipara (%)
Spontaneous	19	21	10
Caesarean	20	23	7
Total	39 (39)	44 (44)	17(17)

Participants completed and returned the questionnaire personally at the hospital.

Age, mode of birth, parity, and educational level were extracted from participants' medical records. Statistical analysis included descriptive statistics (frequencies, percentages, means, and standard deviations) to characterize the sample by mode of delivery, age group, educational level, and parity. Group comparisons were performed using independent samples t-tests and one-way analysis of variance (ANOVA). To examine associations between continuous and ordinal variables (age, educational level, parity) and questionnaire scores, we used Pearson correlation coefficients, as assumptions of approximate normality and linearity were met. Statistical significance was defined as p < 0.05.

Statistical analysis revealed the following mean scores: Spontaneous births: Mean score = 40.74, Caesarean section: Mean score = 42.22 (Table 6).

**Table 6:** Results of the questionnaire by modality of birth

	Spontaneus	Caesarean
Mean Score	40.74	42.22

Comparisons using independent t-tests yielded a statistically significant difference only for the modality of birth (p<0.001). There were no statistically significant differences between age groups or educational levels within each birth modality. ANOVA tests revealed no statistically significant differences among different age groups (p=0.16), educational levels (p=0.052), or parity categories (p=0.31).

Confidentiality of participant data was ensured throughout the study.

## Results

Upon analysis, we observed that patients who underwent cesarean section had a higher quality of sexual life score post-puerperium (42.22) compared to patients who had a spontaneous vaginal birth (40.74). The overall mean ± SD score for the quality of sexual life after puerperium was 41.48 ± 3.83, with a 95% confidence interval (CI) of 40.74 to 42.23 (see Tables 2, 6).

Our findings regarding education level and mode of birth revealed notable results. Patients who underwent a cesarean section and had completed middle school education achieved the highest scores (42.94), whereas those with higher education levels who delivered spontaneously had the lowest scores (40.05). The overall mean ± SD quality of sexual life score by educational level was 41.50 ± 1.07, with a 95% CI of 41.29 to 41.71 (see Tables 4, 7).

**Table 7:** Results of the questionnaire by age

Group	Mean Score	Age 18-25	Age 26-32	Age 33-39
Spontaneous	40.74	40.16	41.33	40.82
Cesarean	42.22	40.82	42.16	42.68

Our data indicated that women with a middle school education who delivered via cesarean section reported the highest sexual function scores (42.94), meanwhile women with higher education levels who delivered vaginally reported the lowest (40.05) (see Table 8).

**Table 8:** Results of the questionnaire by level of education

Group	Mean Score	Education Lower	Education Middle	Education Higher
Spontaneous	40.74	40.53	41.55	40.05
Cesarean	42.22	42.3	42.94	41.65

Our data indicated that multiparous women who delivered by cesarean section reported the highest sexual function scores (43.14), meanwhile primipara women who delivered vaginally reported the lowest (38.94). The overall mean of sexual life score by number of births was 40.74 for the vaginal way and 42.22 for cesarean way (see Table 9).

**Table 9:** Results of the questionnaire by number of births

Group	Mean Score	Primipara	Secundipara	Multipara
Spontaneous	40.74	38.84	41.85	42.4
Cesarean	42.22	41.66	42.47	43.14

Concerning age groups, women aged 33-39 who delivered via cesarean section achieved the highest mean scores (42.68). Conversely, the lowest scores were observed among women aged 18-25 who also underwent cesarean section (40.82). The overall mean ± SD score for quality of sexual life post-puerperium by age group was 40.87 ± 1.63, with a 95% CI of 40.55 to 41.19 (see Tables 3, 6).

Independent sample t-tests were conducted to evaluate the significance of differences between groups. Results indicated that differences in sexual life quality scores based on age and educational levels were not statistically significant, with p-values approaching 1.0 for all comparisons.

ANOVA was used to compare mean scores across multiple groups. The results showed no statistically significant differences based on age groups (p=0.16), educational levels (p=0.052), or parity (p=0.31).

Correlation analysis shown in Table 10 revealed a moderate positive relationship between educational level and postpartum sexual quality-of-life scores (r = 0.211), a weak correlation with parity (r = 0.157), and a weak correlation with age (r = 0.056), based on Pearson correlation coefficients.

**Table 10:** Correlation matrix between way of birth, age, education level, number of births

	Way of Birth	Age	Educational Level	Number of Births	Score
Way of Birth	1.000	0.000	0.000	0.000	0.177
Age	0.000	1.000	0.000	0.000	0.056
Educational Level	0.000	0.000	1.000	0.000	0.211
Number of Births	0.000	0.000	0.000	1.000	0.157
Score	0.177	0.056	0.211	0.157	1.000

## Discussion

As we mentioned in the introduction, the postpartum period is a transformative phase that spans six to eight weeks after childbirth. 

The PISQ-12 is a validated, condition-specific instrument designed to assess sexual function in women with pelvic floor disorders [21]. Endorsed by the International Continence Society with a Grade A recommendation, it serves as a standardized tool for evaluating sexual dysfunction associated with urinary symptoms [22]. The PISQ-12 comprises three domains, behavioral-emotive (items 1-4), physical (items 5-9), and partner-related (items 10-12), and is self-administered using a five-point Likert scale ranging from 0 (always) to 4 (never). Items 1-4 are reverse-scored, with a maximum achievable score of 48, indicating optimal sexual function [23].

Contrary to traditional assumptions that cesarean delivery may confer protection against postpartum sexual dysfunction, current evidence suggests no significant long-term advantage in terms of sexual outcomes when compared to vaginal delivery. Several studies indicate that vaginal birth, including vaginal birth after cesarean (VBAC), is not associated with increased long-term sexual morbidity and may be linked to better sexual outcomes [24]. Perineal trauma, a condition affecting approximately 3% of women in European countries and up to 19% in the United States, is strongly correlated with postpartum dyspareunia, a prevalent form of female sexual dysfunction [25]. Furthermore, severe perineal injury has been consistently shown to delay the resumption of sexual activity and to negatively impact overall sexual function [26]. Our findings corroborate these associations, revealing a statistically significant difference in PISQ-12 scores between women who experienced spontaneous vaginal delivery and those who underwent cesarean section.

Unexpectedly, our data indicated that women with a middle school education who delivered via cesarean section reported the highest sexual function scores, while women with higher education levels who delivered vaginally reported the lowest. These results diverge from established literature, which generally suggests that higher educational attainment is associated with improved sexual health outcomes [27]. While subgroup analysis did not reveal statistically significant differences in PISQ-12 scores across education levels, correlation analysis suggested a moderate influence of educational attainment on postpartum sexual quality of life.

Age-related trends also emerged in our analysis. Women aged 33-39 who delivered via cesarean section reported the highest PISQ-12 scores, whereas those aged 18-25 who also delivered via cesarean reported the lowest scores. One possible explanation is that older women may exhibit greater psychological resilience or better postoperative recovery, contributing to improved sexual outcomes. This aligns with findings by Parks and Choi, who identified younger maternal age at childbirth as a predictor of poorer postpartum sexual quality of life [28]. Nonetheless, other studies have indicated that advanced maternal age is associated with increased obstetric complications, including those related to cesarean delivery [29]. Conversely, some research has found no association between maternal age and postpartum sexual function [30].

These nuanced and sometimes contradictory findings underscore the multifactorial nature of postpartum sexual health and the importance of individualized patient counseling. Further large-scale, longitudinal studies are warranted to better elucidate the complex interplay between delivery mode, maternal age, education level, and sexual well-being.

Our findings suggest that key sociodemographic and obstetric factors, including mode of delivery, maternal age, educational level, and parity, are moderately associated with postpartum sexual function. These results contribute to the growing body of literature emphasizing the multifactorial nature of postpartum sexual health.

Our study employed Pearson correlation to explore the relationships between continuous or ordinal sociodemographic variables and PPQSL-Q scores, under the assumption of linear associations and approximate normality. We acknowledge that future studies with larger samples could apply both Pearson and Spearman coefficients, depending on variable distribution characteristics.

## Conclusions

This study led to the development and initial implementation of a novel, condition-specific PPQSL-Q designed to evaluate sexual function in women during the postpartum period. Developed in the Romanian language and piloted at the University Hospital "Saint Andrew" in Constanța, the questionnaire addresses a significant gap in the assessment tools available to Romanian-speaking women.

The PPQSL-Q is concise, self-administered, and user-friendly, offering a practical resource for both healthcare professionals and postpartum women seeking to assess and address issues related to sexual function. Its implementation has the potential to enhance individualized care and improve overall quality of life during the postpartum period.

To facilitate broader use and international validation, the PPQSL-Q has been translated into English. We anticipate that this tool will serve as a foundation for future research and clinical application in diverse populations, ultimately contributing to improved postpartum care practices.
